# Exercise but Not Supplemental Dietary Tryptophan Influences Heart Rate and Respiratory Rate in Sled Dogs

**DOI:** 10.3390/vetsci7030097

**Published:** 2020-07-23

**Authors:** Emma Thornton, James R. Templeman, Michael Bower, John P. Cant, Graham P. Holloway, Anna K. Shoveller

**Affiliations:** 1 Department of Animal Biosciences, University of Guelph, Guelph, ON N1G 2W1, Canada; ethornto@uoguelph.ca (E.T.); jtemplem@uoguelph.ca (J.R.T.); jcant@uoguelph.ca (J.P.C.); 2 emka TECHNOLOGIES Inc., Falls Church, VA 20166, USA; mbower@emkatech.com; 3 Department of Human Health and Nutritional Sciences, University of Guelph, Guelph, ON N1G 2W1, Canada; ghollowa@uoguelph.ca

**Keywords:** heart rate, respiratory rate, tryptophan, exercise, sled dogs

## Abstract

Tryptophan (Trp), an indispensable amino acid for dogs, is the precursor of serotonin, a neurotransmitter with a variety of effects throughout the body, including the ability to modulate cardiac and pulmonary activity. This study aimed to investigate the effects of a 12-week incremental exercise regimen and supplemental dietary Trp on heart rate (HR) and respiratory rate (RR) in client-owned sled dogs. Sixteen Siberian huskies were randomly allocated to either treatment or control diet groups. Both groups were fed a control diet (Trp to large neutral amino acid ratio of 0.047:1); however, treatment dogs received a Trp supplement to achieve a Trp to large neutral amino acid ratio of 0.075:1. Every three weeks, external telemetry equipment was used to non-invasively measure and record HR and RR at a resting, working, and post-exercise state in a controlled exercise challenge. A mixed model was used to test differences between diet, activity parameter, and week. Dietary Trp supplementation had no effect on HR or RR. Independent of diet, resting, working, post-exercise HR, and time to recover post-exercise HR decreased from week −1 to week 11 (*p* < 0.05). Resting HR had the greatest reduction from week −1 to week 11 (21%, *p* < 0.05). Working RR did not change with exercise (*p* > 0.10), but rRR and postRR decreased from week −1 to week 11 (*p* < 0.05). These data suggest that the exercise regimen the dogs were subjected to may have positively impacted the dogs’ capacity to sustain aerobic exercise, whereas Trp supplementation had no effect on HR or RR.

## 1. Introduction

Sled dogs regularly participate in resistance and aerobic exercise in preparation for, and throughout, their competitive racing season. Their exercise performance can be affected by numerous factors, including ambient temperature [1], feed intake [2], and intensity of exercise [3]. During prolonged exercise, as the workload increases, the oxygen and energy requirements of the muscles increase considerably. As such, heart rate (HR) and respiratory rate (RR) increase to sustain oxygen delivery and help maintain aerobic energy production [4]. Given the integral role of oxygen delivery in aerobic exercise performance, it is well accepted that the cardiorespiratory system is one of the limiting factors for extended bouts of exercise [5,6,7]. Thus, it is important to understand how the cardiorespiratory system can be augmented in order to maximize aerobic potential. In humans and dogs, the respiratory and cardiovascular systems respond to aerobic conditioning through the strengthening of the diaphragm and cardiac hypertrophy, respectively [8,9,10,11]. These adaptations allow for the generation of greater force during inspiration and expiration, as well as ventricular contraction to maximize oxygen intake and delivery [12]. This supports reductions in HR and improvements in respiratory capacity that have been reported following a structured exercise regimen [13,14]. Therefore, by measuring specific changes in parameters such as HR and RR that occur in the dog in response to exercise, we can elucidate how a controlled training regimen may support cardiorespiratory adaptations and performance during a training season.

Dietary adaptations have also been reported to positively affect exercise performance in dogs. Dogs fed diets high in protein and fat (dietary protein > 30%, dietary fat > 40%) have been reported to outperform dogs fed a low-protein, low-fat, and high-carbohydrate diet (dietary protein < 30%, dietary fat < 40%) in areas such as hunting (more successful finds) [15], stamina [16], and decreased risk of injury (8 times lower) [17] during endurance type exercise. The goal of mushers and trainers is to maximize the performance of their dogs, and nutritional solutions may assist and support adaptations to the cardiorespiratory system.

Tryptophan (Trp) is an indispensable amino acid (AA) for dogs and the sole precursor for serotonin (5-hydroxytryptamine, 5-HT), a neurotransmitter that can trigger various physiological responses within the body. The binding of serotonin to specific receptors can elicit effects on HR and RR by either stimulatory or inhibitory mechanisms. However, the central biosynthesis of 5-HT to evoke a response in HR and RR is dependent on the Trp concentration in the central nervous system (CNS) [18,19,20]. In order to be used for central serotonin synthesis, Trp must compete with the large neutral AA (LNAA; valine, leucine, isoleucine, phenylalanine, and tyrosine) for access to blood brain barrier (BBB) transporters [21]. Once in the brain, Trp is initially converted into 5-hydroxytryptophan by tryptophan hydroxylase (TPH), a rate-limiting enzyme that under normal physiological conditions is not fully saturated [22]. Therefore, increasing the Trp:LNAA ratio should increase the likelihood of synthesizing central serotonin which, in turn, may alter cardiac and pulmonary function. The effects of serotonin on pulmonary and vascular responses have been reported in various animal models; however, responses vary depending on the species. Following supraphysiological serotonin injections, cats experienced a decreased HR [23] and dogs an increased HR [24], whereas rats experienced significant changes to RR in the form of apnoea [25], and rabbits had an elevated RR [26]. These responses are a result of direct injections of serotonin, and, to our knowledge, the effect of changes in serotonin status by way of increased dietary Trp supplementation on cardiovascular and respiratory outcomes has not been evaluated in dogs. By utilizing sled dogs, a cohort that is routinely subjected to prolonged bouts of strenuous exercise and who share similar genetics, dietary management, housing, and training regimens, we can examine the effects of Trp supplementation and exercise on HR and RR using fewer animals than if an alternative cohort of healthy adult dogs at maintenance was used. Thus, the objectives of this study was to investigate the effects of 12 weeks of incremental exercise conditioning and an increased Trp:LNAA ratio via supplemental dietary Trp on pre, mid, and post-exercise HR and RR in actively training sled dogs.

## 2. Materials and Methods

### 2.1. Animals and Housing

Sixteen client-owned, domestic Siberian huskies (9 females: 4 intact, 5 spayed; 7 males: 2 intact, 5 neutered), with an average age of 4.8 ± 2.5 years (mean ± standard deviation, SD) and body weight (BW) of 24.3 ± 4.3 kg, were used in the study. Dogs were housed and trained at an off-site facility (Rajenn Siberian Huskies, Ayr, ON, Canada) that had been approved by the University of Guelph’s Animal Care Services. During the study, dogs were housed in free-run, outdoor kennels (3.5–80 square meters) containing anywhere from 2 to 10 dogs each, in which one house per dog was provided for rest and shelter. The study was approved by the Animal Care Committee of the University of Guelph (Animal Use Protocol #4008).

### 2.2. Diets and Study Design

For complete diet and study design, refer to Templeman et al. [27]. In brief, dogs were blocked for age, sex, and BW before being randomly allocated to one of two diet groups: control (Ctl; *n* = 8; 4 males, 3 neutered, 1 intact; 4 females, 2 spayed, 2 intact) or treatment (Trt; *n* = 8; 3 males, 2 neutered, 1 intact; 5 females, 3 spayed, 2 intact). For 2 weeks prior to the study period (weeks −2 and −1), all dogs were acclimated to the dry, extruded Ctl diet (dry matter basis: 4074 kcal/kg metabolizable energy (ME), 94% dry matter (on an as-fed basis), 16% nitrogen-free extract (NFE), 47% crude protein, 25% fat, 0.46% Trp, 1.14 g Trp/Mcal; Champion Petfoods LT., Morinville, AB, Canada) that met or exceeded all National Research Council [28] and Association of American Feed Control Officials [29] nutrient recommendations for adult dogs at maintenance. For additional details regarding the ingredient and nutrient composition of the Ctl diet, refer to Templeman et al. [27]. Dogs in the Ctl group were fed the Ctl diet throughout the entire study period, while Trt dogs were fed the Ctl diet supplemented with dietary Trp (ADM Animal Nutrition, Woodstock, ON, Canada) so as to achieve a Trp:LNAA ratio of 0.075:1 (dry matter basis: 0.73% Trp, 1.80 g Trp/Mcal; Trp:LNAA ratio of Ctl diet was 0.047:1). This ratio was determined with the goal of exceeding both the minimum dietary Trp:LNAA ratios as derived from the National Research Council’s [28] suggested minimum amino acid requirements for adult dogs at maintenance (Trp:LNAA ratio of 0.061:1) as well as the highest Trp:LNAA ratio used when feeding medium or large breed dogs their estimated Trp requirements as determined using indicator amino acid techniques (Trp:LNAA ratio of 0.074:1, medium breed dogs) [30]. All dogs were fed once daily, and all dogs were tethered and fed individually to allow accurate monitoring of food consumption. Any orts were weighed and recorded daily. Throughout the entire trial period, dogs had ad libitum access to fresh water.

### 2.3. Exercise Regimen

For the complete exercise regimen, refer to Templeman et al. [27]. In brief, a 12-week exercise regimen was implemented, with exercise distance increasing incrementally throughout the trial period. However, weather played a role in setting the daily distance. Training consisted of all dogs running on a standard 16-dog gangline that was attached to an all-terrain vehicle (ATV). A pace of approximately 15 km/h was averaged throughout the training period.

Every three weeks, starting on week −1 (weeks −1, 2, 5, 9, 11), one off-day in the dogs’ training schedule (no running) was replaced by an exercise challenge. Dogs were run at a consistent distance at a pace of ~15 km/h in four-dog teams. At week −1, the team of 4 dogs that the owners deemed the least experienced and physically fit were run at a pace of ~15 km/h until any one dog presented with a HR exceeding 300 beats per min (bpm) [31] or displayed any of the predetermined fatigue-associated signs, such as lack of motivation, loose tug line or tight neckline, leaning on gang line, increased salivation, or any other changes deviating from normal exercise behavior. Thereafter, all exercise challenges for all groups of dogs throughout the study (weeks −1, 2, 5, 11) were run at this distance, which was set at 4 km and at a pace of 15 km/h. During each exercise challenge, all dogs were equipped with external telemetry jackets to non-invasively record both HR and RR (emka TECHNOLOGIES, Falls Church, VA, USA).

### 2.4. Telemetry Jackets

Jacketed non-invasive telemetry devices (emkaPACK 4G, emka TECHNOLOGIES, Falls Church, VA, USA) were used to collect the HR and RR sensor data. For non-invasive recordings of HR, electrocardiographic (ECG) readings were obtained from the placement of 4 electrodes on each dog: 2 placed axillar and 2 placed posterior to their right and left scapula (Figure S1). Before placement of electrodes, each attachment site was shaved, cleaned using isopropyl alcohol to remove any lipids on the skin surface, and wiped with skin adhesion wipes (Smith & Nephew, London, UK). To minimize signal noise during a session, electrodes and wires were taped and securely wrapped around the dog’s body to prevent any movement during recording. In addition, an undershirt and jacket were worn by each dog to help secure electrodes and wires into position and to reduce exposed wires which were likely to be chewed, caught on objects, or induce noise. Heart rate data were recorded every 10 s during the exercise challenges, using the IOX software from emka TECHNOLOGIES (emka TECHNOLOGIES, Falls Church, VA, USA). Only HRs exceeding an 80% success rate as well as appropriate ECG signals through examination of the ECG trace were used in the current study, to distinguish heartbeats from artifacts. This technique has been previously validated in dogs [32]. In addition, discrete stages of HR were recorded, which comprised resting HR (rHR), working HR (wHR), post-exercise HR (postHR), and totalHR (average of all activity levels combined). Each HR stage was recorded for all dogs at each exercise challenge. In addition, the time required to reach a post-exercise HR (tpostHR) was also determined.

Non-invasive measurements and recordings of RR were obtained using respiration inductance plethysmography (RIP), which utilizes custom fit respiratory bands placed around the dogs’ thoraxes to measure the expansion and retraction of the thorax during a breathing cycle (Figure S1). Respiratory rate data were recorded, and mean values were derived for every 5 breaths. Breath detection thresholds were established for each subject by observing the signal amplitudes of the RIP sensors during peak inspiration and expiration and setting the detection thresholds to a level sufficiently above the noise floor. Additionally, the data were filtered using a digital bandpass filter with a 0.1 to 30 Hz @-3 dB. The band pass filter was used to allow breathing frequencies between 0.1 and 30 Hz to be detected and recorded, while any other frequencies not within this range were attenuated. In addition, only RRs exceeding an 80% success rate were used in the current study, to distinguish breaths from artifacts. Resting RR (rRR), working RR (wRR), post exercise RR (postRR), and totalRR (average of all activity levels combined) were recorded at each challenge. In addition, the time required to reach a post-exercise RR (tpostRR) was recorded.

All resting data were collected when the dogs were lying quietly in a secluded room with limited dog-to-dog or human-to-dog interaction for approximately 1 h to allow their HR and RR to decrease to resting. Following resting data collection, dogs were put into harnesses and placed on the 4-dog gangline, where the challenge run commenced and where wHR and wRR were recorded. Once the challenge ceased, dogs were immediately watered and remained on the gangline for 1 h to continue gathering HR and RR data until postHR and postRR were obtained, from which tpostHR and tpostRR data were determined.

### 2.5. Statistical Analysis

All statistical analyses were performed with the Statistical Analysis System (SAS v. 9.4; SAS Institute Inc., Cary, NC, USA). Heart rate and RR data were analyzed for outliers by removing the data points that exceeded a HR greater than 350 bpm and lower than 10 bpm. For RR, data points greater than 200 breaths per min and lower than 5 breaths per min were also considered outliers and subsequently removed. In addition, the periods during the exercise challenges when a 4-dog team was stopped were removed as these interruptions interfered with the dogs’ ability to maintain a wHR or wRR. These stops included instances of defecation and urination, as well as distractions such as wildlife which caused the dogs to alter their course and/or stop the run briefly. In addition, as exercise challenges were run on back roads, stops were made when crossing streets and turning around at the halfway point of the run. Following the outlier removal, a TRANSREG procedure was used to transform the data before HR and RR were analyzed as a repeated measure over time using PROC GLIMMIX. When fixed effects were significant, means were compared using the Tukey Honestly Significant Difference (HSD) test. Dog was treated as a random effect, and activity (resting, working, post exercise, and time to post-exercise), week, and diet group were treated as fixed effects. Time to post-exercise HR and RR was determined from the point at which exercise stopped following an exercise challenge until a plateaued HR and RR was measured using a TRANSREG procedure. The time point from immediate cessation of exercise up until the plateau was achieved was used, and a mean time was calculated for recovery. A PROC CORR was used where appropriate to assess the strength of a linear relationship between all activity parameters (rHR, rRR, wHR, wRR, postHR, postRR, tpostHR, tpostRR), temperature (°C), and week. Statistical significance was declared at *p* ≤ 0.05 and trends at *p* ≤ 0.10.

## 3. Results

During data analysis, 17% (43/256) of the HR observations and 15% of RR observations (29/192) were removed due to either software malfunctions, which caused inaccurate readings, or interruptions during the challenge run, which altered with wHR or wRR. Two dogs were removed from the trial (one Ctl on week 7; one Trt on week 9) due to exercise-related injuries. All data collected from these dogs until their removal were included in the analyses. Due to inclement weather, adjustments were made to the proposed 12-week incremental training regimen, including the removal of the week 9 exercise challenge and a descaling of the daily running duration by approximately half (maximum distance run was 34 km by week 12). Environmental temperatures for the four exercise challenges were recorded using a digital thermometer (Gain Express, Hong Kong, China). The mean ambient temperature for each of the four challenge days were as follows: 5 °C (week −1), 4 °C (week 2), −1 °C (week 5), 2 °C (week 11). For information regarding food intake, run distance, BW, and additional details regarding the training schedule, refer to Templeman et al. [27]. In brief, food intake, run distance, and BW did not differ between diet groups, but all differed between weeks. Run distance followed an incremental increase until week 7, when we were forced to return to distances equivalent to week 4. Food intake was greatest at weeks 6 and 7, before our runs were descaled and diet intakes were correspondingly reduced to week 5 levels. Body weight was lowest from week 3 to week 7, but baseline body weights were maintained from weeks 8 to 11.

### 3.1. Heart Rate

**Figure 1:**
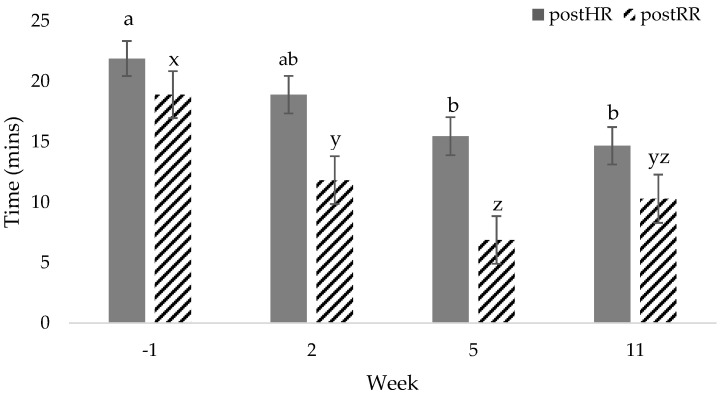
Total mean time ± standard error (SEM) to reach post-exercise heart rate (postHR) and respiratory rate (postRR) after exercise challenge on weeks −1, 2, 5, 11 in sled dogs running at a pace of 15 km/h for 4 km. ^a,b,c^ HR or RR values with different superscripts are different among weeks (*p* < 0.05).

No differences were found between the diet groups for rHR, wHR, postHR, or tpostHR (*p* > 0.10); therefore, these data were pooled to report the effect of exercise. For all activity parameters (resting, working, and recovery post-exercise), HR significantly decreased by week 11 when compared to week −1 values (*p* < 0.05, Table 1). Resting HR had the greatest reduction in proportion to baseline values over time (21%, *p* < 0.05), while wHR had the smallest reduction, at 17% (*p* < 0.05; Table 2). Time required to recover HR post-exercise was significantly decreased by week 5 (*p* <0.05; Figure 1) but had no difference thereafter (*p* > 0.10). Environmental temperature was positively correlated with rHR (*r* = 0.341, *p* < 0.05) but was not correlated with any other activity parameter (*p* > 0.10). Run distance was negatively correlated with rHR (*r* = −0.441, *p* < 0.05) and postHR (*r* = −0.361, *p* < 0.05) but had a tendency to be correlated with wHR (*r* = −0.274, *p* = 0.07). No differences were found when comparing sex, BW, or age with rHR, wHR, and postHR over weeks (*p* > 0.10).

### 3.2. Respiratory Rate

No differences were found between diet group and rRR, wRR, or postRR (*p* > 0.10); therefore, these data were pooled to report the effects of exercise. Control dogs had a more rapid recovery from baseline (*p* < 0.05), while Trt dogs took 3 weeks longer to recover (*p* < 0.05; Table 3); however, there was no diet effect (*p* > 0.10; Table 3). No other differences were observed between diet groups and tpostRR (*p* > 0.10); as such, the diet groups were pooled to examine the effects of training. Respiratory rate was greatest at week −1 compared to week 11 for both resting and post-exercise activity levels (*p* < 0.05; Table 4). There was no change in wRR over time (*p* > 0.10; Table 4). Time to post-exercise RR decreased over time and was negatively correlated with week (r = −0.354, *p* < 0.05). No differences were observed in tpostRR between weeks 2 and 11 or weeks 5 and 11 (*p* > 0.10; Figure 1). Environmental temperature was positively correlated with rRR (r = 0.407, *p* < 0.05) and postRR (r = 0.457, *p* < 0.05) but was not correlated with wRR (*p* >0.10). Run distance was negatively correlated with rRR (r = −0.409, *p* < 0.05) and postRR (r = −0.481, *p* < 0.05) but was not correlated with wRR (*p* > 0.10). No differences were found when comparing age, BW, or sex with rRR, wRR, or postRR over weeks (*p* > 0.10).

## 4. Discussion

The data presented herein indicate that for mid-distance training sled dogs, 12 weeks of dietary Trp supplementation had little to no effect on HR or RR. However, the conditioning regimen that the dogs were subjected to may have resulted in a reduction in HR and RR as well as a more rapid recovery to a resting state following exercise.

Our laboratory previously reported that Trp supplementation, at levels necessary to achieve a dietary Trp:LNAA ratio of 0.075:1, resulted in an increased 5-HT level in Trt dogs by week 11 [27]. In the current study, dogs fed the Ctl diet presented with more rapid RR recovery times by week 2, whereas dogs fed the diet supplemented with Trp recovered to the same extent by week 5. However, there was no diet effect on RR recovery, suggesting that Trp supplementation did not affect respiratory parameters during the exercise regimen. As previous research reports serotonin increases HR and RR in dogs [24,33], the minimal effects presented in the current study suggest that the increased Trp concentrations identified in serum by week 5 [27] may not have been to such an extent as to influence 5-HT or cardiac and respiratory rates. In addition, no changes were observed in HR or RR during week 11 when 5-HT concentrations were increased [27]. It has been previously hypothesized that the intensity of aerobic exercise influences the sensitivity and downregulation of 5-HT receptors in various animal models [34,35]. As the dogs in the current study were subjected to a training regimen that accumulated a total run distance of ~1200 km, the possibility of the downregulation of 5-HT receptors by week 11 of the current study could support the limited changes seen. Therefore, as 5-HT levels in serum were not increased until the final week of the study (week 11), future research is warranted to investigate the effects of HR and RR over an extended period of time. In addition, Trp metabolism is influenced by two major pathways, the kynurenine and the serotonergic pathways. The kynurenine pathway, which consumes more of the available free Trp than the serotonergic pathway [36], is involved in ATP production, with oxidation as the main outcome and nicotinamide adenine dinucleotide (NAD) as a minor outcome [22]. Upregulation of the kynurenine pathway by various factors during exercise (i.e., steroid hormones and inflammatory cytokines) [37,38,39] could explain the lack of treatment effect on HR and RR, where the majority of supplemented Trp was used for protein synthesis and energy production through the kynurenine pathway rather than for cardiorespiratory modifications. However, the current study did not measure kynurenine pathway metabolites, steroid hormones, or inflammatory cytokines and, as such, cannot definitively say that Trp metabolism was largely involved in the kynurenine pathway over the serotonergic pathway. Regardless, independent of Trp supplementation, the reduction in HR and RR following a conditioning regimen is consistent with previous studies in dogs, indicating that whole-body physiological adaptations may have occurred [9,40,41].

The reductions in resting, working, and post-exercise HR observed in the present study are comparable to previous research examining both sled dogs and mongrel dogs [8,9,42]. For example, Stepien and colleagues [9] noted a 15% reduction in rHR in Alaskan huskies following 5 months of aerobic training, while Tipton et al. [43] reported a 40-bpm difference in wHR following 10 weeks of aerobic training in mongrel dogs. In addition, Billman and Kukielka [44] examined the post-exercise HR of 10 dogs before and after 10 weeks of aerobic training and reported an overall decrease in HR and more rapid recovery to resting HR following exercise. These changes were attributed to adaptations in autonomic control and cardiac morphology. As a 21% decrease in rHR, 35-bpm reduction in wHR, and quicker recovery times post-exercise were observed in the current study, it is likely that these dogs also underwent physiological modifications.

Previous studies investigating cardiorespiratory measurements following various training levels (8 months to 5 years) in humans, reported decreases in breathing frequency when compared to sedentary and lesser trained counterparts [45,46]. In the current study, decreases in RR were observed at both a resting and post-exercise state, whereas no changes were reported in wRR. However, due to adjustments that were made to the training regimen in the current study, the lack of differences observed in wRR may have been attributed to a reduction in training load. As descaling of the training regimen took place at week 7, the maximum distance run by week 11 was equivalent to the distance run by week 4 (34 km). As such, the incremental training regimen was no longer implemented from week 7 onward. Therefore, the duration of the training program may not have been to the extent required to induce physiological adaptations [47]. However, as wHR decreased, our data may suggest that the respiratory system requires consistent training in order to adapt to exercise, as it appears to be more sensitive to a reduced training workload. In support of this, Amory et al. [48] reported that, following a 4-week training program in ponies, 2 weeks of detraining caused RR to vary in breathing frequency at both exercise and recovery states, while no such changes were reported in HR. This study, as well as the current study, underpin the importance of continuous training for the maintenance of training-induced adaptations. However, even though wRR did not change following a conditioned state, rRR, postRR, and tpostRR decreased with 12 weeks of training, suggesting a possible training effect. However, independent of exercise-induced decreases in HR and RR, habituation to novel objects can also affect cardiorespiratory parameters [49]. As no adaptation period was provided to the dogs in the current study for exercise challenge days, the decreases in HR and RR could indicate a possible habituation to the novel equipment and set-up over time, rather than to the exercise regimen itself. Thus, providing a period of adaptation to the equipment prior to the study measures, as well as investigating the effects of habituation to the equipment, in both exercised and sedentary control groups of dogs could be beneficial in providing further insight.

Since sled dogs are known to train and race in cold climates, both the environment in which their exercise takes place and their internal temperature could influence cardiorespiratory outcomes [50,51]. Dogs do not dissipate heat in the same manner as humans; rather, dogs rely primarily on hyperventilation (panting) for heat dissipation during exercise [52]. In the current study, both HR and RR were found to be correlated with temperature, indicating that as ambient temperature increased, so too did the beat and breathing frequency. However, as the warmest weather was measured at week −1 of the study (5 °C), the correlation between HR and RR with temperature could also be attributed to the unconditioned state of the dogs. This can also be seen as week 5 was recorded with the lowest temperature at −1 °C, whereas week 11 was 2 °C, and no differences between these two exercise challenges were observed, suggesting that environmental temperature may have had little effect on HR and RR.

The authors do acknowledge that there were limitations to this study that should be noted. Foremost, with regard to cardiorespiratory parameters, only HR and RR were measured; however, other cardiorespiratory parameters, such as heart rate variability, stroke volume, maximal oxygen consumption, minute ventilation, and tidal volume, could be monitored alongside HR and RR to attempt to elucidate the changes occurring during a training period. In addition, no adaptation period to the equipment was given prior to the start of the study or collection of body temperatures during exercise challenges, which may have had a possible influence on HR and RR. In addition, the descaling of the incremental training regimen and removal of an exercise challenge day may have impacted our measured parameters. Future studies are warranted to investigate the influence of an incremental training regimen and dietary Trp supplementation on sled dogs, using additional cardiorespiratory outcomes while also considering the effects of body temperature, environmental temperature, and habituation responses on HR and RR.

## 5. Conclusions

Overall, the aim of this study was to evaluate the effects of dietary Trp supplementation as well as a 12-week training regimen on the outcomes of HR and RR in client-owned Siberian huskies training for mid-distance races. The findings of the current study suggest that repetitive endurance exercise potentially promotes the adaptation of both cardiac and pulmonary systems, as evident through reductions in mean HR, RR, and the time required to achieve a recovery state post-exercise. These data indicate that after 12 weeks of exercise conditioning, independent of Trp supplementation, rHR, rRR, wHR, postHR, and postRR decreased from week −1. In addition, within the constraints of this study design, there were no effects of Trp supplementation on HR and RR, but future research regarding Trp supplementation, serotonin status, an incremental training regimen, and various cardiorespiratory parameters is warranted. In addition, familiarity with the equipment as well as body temperature could also have influenced our results and should be considered. However, this research suggests that when developing a training regimen, maintenance of incremental increases in intensity and duration may support performance in sled dogs during mid-distance races.

## Figures and Tables

**Table 1:** Least square mean heart rate (beats per minute) at rest, work, and post-exercise on week −1, 2, 5, and 11 challenges in sled dogs running at a pace of 15 km/h for 4 km.

Activity Parameter ^1^	Exercise Challenge				SEM ^2^	*p* Value		
	Week −1	Week 2	Week 5	Week 11		Week	Diet	Week x Diet
totalHR, bpm ^3^	194 ^a^	182 ^b^	179 ^b,c^	168 ^c^	3	<0.01	0.10	0.57
rHR, bpm	91 ^a^	79 ^b^	71 ^b^	68 ^b^	4	<0.01	0.38	0.57
wHR, bpm	270 ^a^	249 ^a,b^	261 ^a,b^	234 ^b^	9	<0.01	0.10	0.17
postHR, bpm	110 ^a^	101 ^a,b^	95 ^b^	92 ^b^	4	<0.01	0.55	0.85

^1^ TotalHR, total mean heart rate; rHR, resting heart rate; wHR, working heart rate; postHR, post-exercise heart rate. ^2^ SEM, standard error of the mean, *n* = 16 for rHR (weeks −1, 2, and 5), *n* = 14 for rHR (week 11), *n* = 16 for wHR (week −1), *n* = 12 for wHR (week 2), *n* = 7 for wHR (week 5), *n* = 10 for wHR (week 11), *n* = 16 for postHR (week −1), *n* = 14 for postHR (weeks 2, 5, and 11). ^3^ bpm, beats per minute. ^a,b,c^ Values in a row with different superscripts are different (*p* < 0.05). x Shows the interaction effect between week and diet.

**Table 2:** Change from baseline (week −1) as proportion of heart rate at rest, work, and post-exercise on week −1, 2, 5 and 11 challenges in sled dogs running at a pace of 15 km/h for 4 km.

Activity Parameter ^1^	Exercise Challenge				SEM ^2^	*p* Value		
	Week −1	Week 2	Week 5	Week 11		Week	Diet	Week x Diet
totalHR, % of total	194	−10 *	−12 *	−17 *	2	<0.01	0.10	0.57
rHR, %	91	−8	−20 *	−21 *	4	<0.01	0.30	0.81
wHR, %	270	−15 *	−5	−17 *	3	<0.01	0.18	0.06
postHR, %	110	−11	−16	−19 *	4	<0.01	0.87	0.88

^1^ TotalHR, total mean heart rate; rHR, resting heart rate; wHR, working heart rate; postHR, post-exercise heart rate. ^2^ SEM, standard error of the mean, *n* = 16 for rHR (weeks −1, 2, and 5), *n* = 14 for rHR (week 11), *n* = 16 for wHR (week −1), *n* = 12 for wHR (week 2), *n* = 7 for wHR (week 5), *n* = 10 for wHR (week 11), *n* = 16 for postHR (week −1), *n* = 14 for postHR (weeks 2, 5, and 11). * Values in a row with an asterisk are different from baseline (*p* ≤ 0.05). x Shows the interaction effect between week and diet.

**Table 3:** Least square mean time to recovery respiratory rate (mins) post-exercise for both diet groups on week −1, 2, 5, and 11 challenges in sled dogs running at a pace of 15 km/h for 4 km.

Diet Group ^1^	Exercise Challenge				SEM ^2^	*p* Value		
	Week −1	Week 2	Week 5	Week 11		Week	Diet	Week x Diet
Trt, mins	19 ^a^	16 ^a^	6 ^b^	12 ^a,b^	2	<0.01	0.37	0.06
Ctl, mins	18 ^a^	7 ^b^	7 ^b^	8 ^b^	2	<0.01		

^1^ Trt, treatment; Ctl, control. ^2^ SEM, standard error of the mean, *n* = 7 for Trt (weeks −1, 2, and 11), *n* = 8 for Trt (week 5); *n* = 8 for Ctl (week −1), *n* = 7 for Ctl (week 2), *n* = 6 for Ctl (weeks 5 and 11). ^a,b,^^c^ Values in a row with different superscripts are different (*p* ≤ 0.05). x Shows the interaction effect between week and diet.

**Table 4:** Least square mean respiratory rate (breathing frequency) at rest, work, and post-exercise on week −1, 2, 5, and 11 challenges in sled dogs running at a pace of 15 km/h for 4 km.

Activity Parameter ^1^	Exercise Challenge				SEM ^2^	*p* Value		
	Week −1	Week 2	Week 5	Week 11		Week	Diet	Week x Diet
totalRR, bf ^3^	75 ^a^	73 ^a,b^	69 ^b^	68 ^b^	1	<0.01	0.36	0.81
rRR, bf	23 ^a^	19 ^a,b^	15 ^c^	16 ^b,c^	1	<0.01	0.66	0.12
wRR, bf	176 ^a^	172 ^a^	174 ^a^	169 ^a^	4	0.50	0.86	0.74
postRR, bf	27 ^a^	26 ^a^	18 ^b^	20 ^b^	1	<0.01	0.17	0.55

^1^ totalRR, total mean respiratory rate; rRR, resting respiratory rate; wRR, working respiratory rate; postRR, post-exercise respiratory rate. ^2^ SEM, standard error of the mean, *n*= 14 for rRR (week −1), *n* = 16 for rRR (weeks 2, 5), *n* = 13 for rRR (week 11), *n* = 15 for wRR (week −1), *n* = 14 for wRR (week 2), *n* = 8 for wRR (week 5), *n* = 9 for wRR (week 11), *n* = 15 for postRR (week −1), *n* = 14 for postRR (weeks 2 and 5), *n* = 13 for postRR (week 11). ^3^ bf, breathing frequency (breaths per minute). ^a,b,c^ Values in a row with different superscripts are different (*p* ≤ 0.05). x Shows the interaction effect between week and diet.

## Supplementary Materials

The following are available online at https://www.mdpi.com/2306-7381/7/3/97/s1. Figure S1: Respective electrode placement for non-invasive recording of HR (A) and placement of a custom fit respiratory band on the dogs’ thoraxes to non-invasively record RR (B) during rest, working, and post-exercise training levels.

## References

[1] Robbins P., Ramos M., Zanghi B., Otto C., Robbins P. (2017). Environmental and physiological factors associated with stamina in dogs exercising in high ambient temperatures. Front. Vet. Sci..

[2] Orr N. (1966). The feeding of sledge dogs on Antarctic expeditions. Br. J. Nutr..

[3] Taylor R. (1957). The work output of sledge dogs. J. Physiol..

[4] Rovira S., Munoz A., Benito M. (2008). Effect of exercise on physiological, blood and endocrine parameters in search and rescue-trained dogs. Vet. Med. Czech..

[5] Sonetti D., Wetter T., Pegelow D., Dempsey J. (2001). Effects of respiratory muscle training versus placebo on endurance exercise performance. Respir. Physiol..

[6] Hall J.E. (2011). Guyton and Hall: Textbook of Medical Physiology.

[7] Dunham C., Harms C. (2012). Effects of high-intensity interval training on pulmonary function. Eur. J. Appl. Physiol..

[8] Constable P., Hinchcliff K., Olson J., Hamlin R. (1994). Athletic heart syndrome in dogs competing in a long-distance sled race. J. App. Physiol..

[9] Stepien R., Hinchcliff K., Constable P., Olson J. (1998). Effect of endurance training on cardiac morphology in Alaskan sled dogs. J. Appl. Physiol..

[10] Pluim B., Zwinderman A., van Der Laarse A., van Der Wall E., Pluim B. (2000). The athlete’s heart. A meta-analysis of cardiac structure and function. Circulation.

[11] Downey A., Chenoweth L., Townsend D., Ranum J., Ferguson C., Harms C. (2007). Effects of inspiratory muscle training on exercise responses in normoxia and hypoxia. Respir. Physiol. Neurobiol..

[12] Hodgson D., Hodgson D., Harrington McKeever K., McGowan C. (2014). Chapter 11-The cardiovascular system: Anatomy, physiology, and adaptations to exercise and training. The Athletic Horse.

[13] Sugawara J., Murakami H., Maeda S., Kuno S., Matsuda M. (2001). Change in post-exercise vagal reactivation with exercise training and detraining in young men. Eur. J. Appl. Physiol..

[14] Huang G., Wang R., Chen P., Huang S., Donnelly J., Mehlferber J. (2016). Dose–response relationship of cardiorespiratory fitness adaptation to controlled endurance training in sedentary older adults. Eur. J. Prev. Cardiol..

[15] Davenport G., Kelley E., Altom E., Lepine A. (2001). Effect of diet on hunting performance of English pointers. Vet. Ther..

[16] Kronfeld D. (1973). Diet and the performance of racing sled dogs. J. Am. Vet. Med. Assoc..

[17] Reynolds A., Reinhart D., Carey D., Simmerman D., Frank D., Kallfelz F. (1999). Effect of protein intake during training on biochemical and performance variables in sled dogs. Am. J. Vet. Res..

[18] Dalton D. (1986). The cardiovascular effects of centrally administered 5-hydroxytryptamine in the conscious normotensive rat. J. Auton. Pharmacol..

[19] Lalley P., Bischoff A., Richter D., Lalley P. (1994). 5-HT-1A receptor-mediated modulation of medullary expiratory neurones in the cat. J. Physiol..

[20] Berger M., Gray J., Roth B. (2009). The expanded biology of serotonin. Annu. Rev. Med..

[21] Ruddick J., Evans A., Nutt D., Lightman S., Rook G., Lowry C. (2006). Tryptophan metabolism in the central nervous system: Medical implications. Expert Rev. Mol. Med..

[22] O’Mahony S., Clarke G., Borre Y., Dinan T., Cryan J. (2015). Serotonin, tryptophan metabolism and the brain-gut-microbiome axis. Behav. Brain Res..

[23] Jacobs L., Comroe J., Jacobs L. (1971). Reflex apnea, bradycardia, and hypotension produced by serotonin and phenyldiguanide acting on the nodose ganglia of the cat. Circ. Res..

[24] Eckstein R., Shintani F., Rowen H., Shimomura K., Oya N. (1971). Identification of left coronary blood supply of aortic bodies in anesthetized dogs. J. Appl. Physiol..

[25] Mitchell H., Tomlin J., Ward R. (1984). Reflex changes in respiration and heart rate evoked by intravenous and left ventricular injection of 5-HT and capsaicin in anaesthetized rats: A comparison of mechanisms. Lung.

[26] Iovino L., Mutolo D., Cinelli E., Contini M., Pantaleo T., Bongianni F. (2019). Breathing stimulation mediated by 5-HT1A and 5-HT3 receptors within the preBötzinger complex of the adult rabbit. Brain Res..

[27] Templeman J.R., Thornton E., Cargo-Froom C., Squires E.J., Swanson K.S., Shoveller A.K. (2020). Effects of incremental exercise and dietary tryptophan supplementation on the amino acid metabolism, serotonin status, stool quality, fecal metabolites, and body composition of mid-distance trained sled dogs. J. Anim. Sci..

[28] National Research Council (2006). Nutrient Requirements of Dogs and Cats.

[29] Association of American Feed Control Officials (2016). AAFCO Manual.

[30] Templeman J.R., Mansilla W.D., Fortener L., Shoveller A.K. (2019). Tryptophan requirements in small, medium, and large breed adult dogs using the indicator amino acid oxidation technique. J. Anim. Sci..

[31] Van Citters R., Franklin D. (1969). Cardiovascular performance of Alaska sled dogs during exercise. Circ. Res..

[32] Prior H., McMahon N., Schofield J., Valentin J. (2009). Non-invasive telemetric electrocardiogram assessment in conscious beagle dogs. J. Pharmacol. Tox. Met..

[33] Zucker I., Cornish K. (1980). Reflex cardiovascular and respiratory effects of serotonin in conscious and anesthetized dogs. Circ. Res..

[34] Chennaoui M., Grimaldi B., Fillion M., Bonnin A., Drogou C., Fillion G., Guezennec C. (2000). Effects of physical training on functional activity of 5-HT1B receptors in rat central nervous system: Role of 5-HT-moduline. N-S Arch. Pharmacol..

[35] Jakeman P., Hawthorn J., Maxwell S., Kendall M., Holder G. (1994). Evidence for downregulation of hypothalamic 5-hydroxytryptamine receptor function in endurance-trained athletes. Exp. Physiol..

[36] Bender D. (1983). Biochemistry of tryptophan in health and disease. Mol. Aspects Med..

[37] Tremblay M., Copeland J., Van Helder W. (2004). Effect of training status and exercise mode on endogenous steroid hormones in men. J. Appl. Physiol..

[38] Suzuki K., Peake J., Nosaka K., Okutsu M., Abbiss C., Surriano R., Bishop D., Quod M., Lee H., Martin D. (2006). Changes in markers of muscle damage, inflammation and HSP70 after an Ironman triathlon race. Eur. J. Appl. Physiol..

[39] Comassi M., Vitolo E., Pratali L., Del Turco S., Dellanoce C., Rossi C., Santini E., Solini A. (2015). Acute effects of different degrees of ultra-endurance exercise on systemic inflammatory responses. Intern. Med. J..

[40] Ordway G., Charles J., Randall D., Billman G., Wekstein D. (1982). Heart rate adaptation to exercise training in cardiac-denervated dogs. J. Appl. Physiol..

[41] Billman G., Cagnoli K., Csepe T., Li N., Wright P., Mohler P., Fedorov V. (2015). Exercise training-induced bradycardia: Evidence for enhanced parasympathetic regulation without changes in intrinsic sinoatrial node function. J. Appl. Physiol..

[42] Wyatt H., Mitchell J., Wyatt H. (1974). Influences of physical training on the heart of dogs. Circ. Res..

[43] Tipton C., Carey R., Eastin W., Erickson H. (1974). A submaximal test for dogs: Evaluation of effects of training, detraining, and cage confinement. J. Appl. Physiol..

[44] Billman G., Kukielka M. (2007). Effect of endurance exercise training on heart rate onset and heart rate recovery responses to submaximal exercise in animals susceptible to ventricular fibrillation. J. Appl. Physiol..

[45] Boussana A., Hue O., Matecki S., Galy O., Ramonatxo M., Varray A., Le Gallais D. (2002). The effect of cycling followed by running on respiratory muscle performance in elite and competition triathletes. Eur. J. Appl. Physiol..

[46] Di Paco A., Dubé B., Laveneziana P. (2017). Changes in ventilatory response to exercise in trained athletes: Respiratory physiological benefits beyond cardiovascular performance. Arch. Bronconeumol..

[47] Blomqvist C., Saltin B. (1983). Cardiovascular adaptations to physical training. Annu. Rev. Physiol..

[48] Amory H., Art T., Lekeux P., Equine Research Funds Asbl (1988). Effects de l’entraînement sur la fonction cardio-respiratoire et sur la température rectale chez le poney; Effects of training and detraining on heart rate, ventilation and thermoregulation during a standardized treadmill exercise in ponies: A preliminary study. J. Anns. Méd. Vét..

[49] Leiner L., Fendt M. (2011). Behavioural fear and heart rate responses of horses after exposure to novel objects: Effects of habituation. Appl. Anim. Behav. Sci..

[50] Saursaunet V., Norges Teknisk-Naturvitenskapelige Universitet F. (2010). Effect of Ambient Temperature on Endurance Performance in Cross Country Skiers. Master’s Thesis.

[51] Kruk B., Kaciuba-Uściłko H., Nazar K., Greenleaf J., Kozłowski S. (1985). Hypothalamic, rectal, and muscle temperatures in exercising dogs: Effect of cooling. J. Appl. Physiol..

[52] Lewis S., Foster R. (1976). Effect of Heat on Canines and Felines. Iowa State Univ. Vet..

