# Supplementary material for: Exercise but Not Supplemental Dietary Tryptophan Influences Heart Rate and Respiratory Rate in Sled Dogs
Source: Vet Sci. 2020 Jul 23;7(3):97. doi: 10.3390/vetsci7030097 (PMC7559096; doi:10.3390/vetsci7030097)
Supplement: Supplementary file 1 [file vetsci-07-00097-s001.pdf]

veterinary sciences

MDPI

Exercise but Not Supplemental Dietary Tryptophan Influences Heart Rate and Respiratory Rate in Sled Dogs

Emma Thornton, James R. Templeman, Michael Bower, John P. Cant, Graham P. Holloway and Anna K. Shoveller

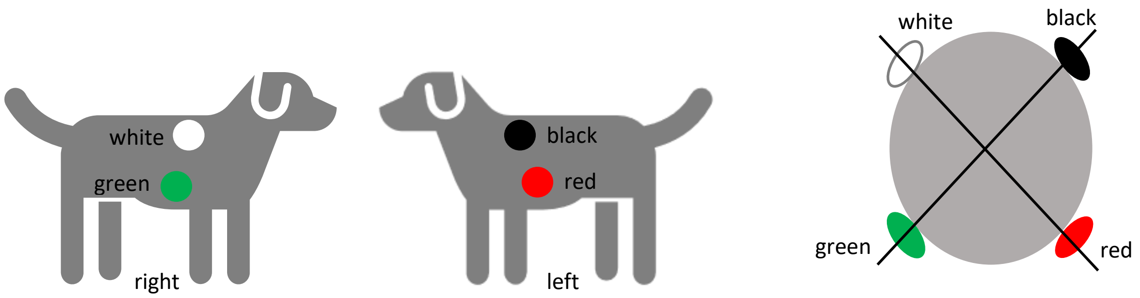

(**A**)

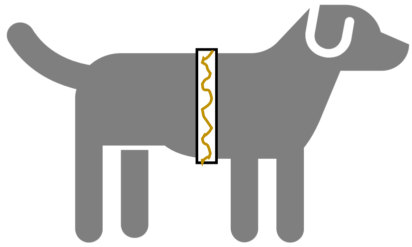

(**B**)

Figure S1. Respective electrode placement for non-invasive recording of HR (**A**) and placement of a custom fit respiratory band on the dogs' thoraxes to non-invasively record RR (**B**) during rest, working, and post-exercise training levels.

© 2020 by the authors. Licensee MDPI, Basel, Switzerland. This article is an open access article distributed under the terms and conditions of the Creative Commons Attribution (CC BY) license (http://creativecommons.org/licenses/by/4.0/).
